# Financial Ties, Market Structure, Commercial Prices, and Medical Director Compensation in Dialysis

**DOI:** 10.1001/jamahealthforum.2025.2659

**Published:** 2025-06-18

**Authors:** Xuyang Xia, Wanrong Deng, Paul J. Eliason, Riley J. League, Ryan C. McDevitt, James W. Roberts, Heather Wong

**Affiliations:** 1 Department of Economics, Trinity College of Arts and Sciences, Duke University, Durham, North Carolina; 2 David Eccles School of Business, University of Utah, Salt Lake City; 3 National Bureau of Economic Research, Cambridge, Massachusetts; 4 Gies College of Business, University of Illinois Urbana-Champaign, Champaign; 5 Duke University, Durham, North Carolina; 6 Fuqua School of Business, Duke University, Durham, North Carolina; 7 Department of Economics, University of Michigan, Ann Arbor

## Abstract

**Importance:**

Growing consolidation in the dialysis industry has raised concerns about market power and the potential need for antitrust enforcement, but a lack of data previously prevented a systematic analysis of these issues.

**Objective:**

To document the consolidation of dialysis facilities through chain ownership and vertical integration between dialysis facilities and physicians, as well as study associations between consolidation and commercial prices for outpatient hemodialysis and medical director compensation.

**Design, Setting, and Participants:**

This economic evaluation combined data from Freedom of Information Act requests, Medicare claims, cost reports, commercial prices, and physician registries to conduct a retrospective study of market structure, compensation, and prices in the dialysis industry from 2005 to 2019. Those evaluated include dialysis facilities in the US, physicians involved with dialysis for patients enrolled in Medicare, and patients with end-stage renal disease enrolled in Medicare or one of the commercial insurers in claims data provided by the Health Care Cost Institute. Data were analyzed from April 2024 to April 2025.

**Exposure:**

Chain ownership, physician ownership, and medical directorship of dialysis facilities in the US from 2005 to 2019.

**Main Outcomes and Measures:**

Chain ownership of dialysis facilities, share of population living in markets with different levels of chain ownership, share of facilities with physician owners, levels of medical director compensation, and commercial prices for outpatient hemodialysis in markets with different numbers of chain- and physician-owned facilities.

**Results:**

Between 2005 and 2019, the market share of DaVita and Fresenius increased from 59.1% to 77.1%, with 32.5% of the national population living in a hospital service area (HSA) without access to a dialysis facility other than these 2 chains in 2019. The share of facilities with a physician owner increased from 11.4% to 29.1%. Markets with only 1 large chain had $495.08 (95% CI, $371.21-$619.85) higher mean commercial prices for outpatient hemodialysis and $564.56 (95% CI, $429.03-$700.10) higher medical director compensation per patient than markets that did not have large chain facilities.

**Conclusions and Relevance:**

In this economic evaluation, market concentration in the US dialysis industry increased from 2005 to 2019 and was associated with higher commercial prices for outpatient hemodialysis. Physician ownership of facilities also increased, and medical directors received higher pay in markets where large chains operate.

## Key Points

**Question:**

How did facility ownership in the US dialysis industry evolve between 2005 and 2019, and what is its association with commercial prices and medical director compensation?

**Findings:**

In this economic evaluation, between 2005 and 2019, the US dialysis industry consolidated both horizontally and vertically, with the share of facilities operated by the 2 largest dialysis chains, DaVita and Fresenius, increasing from 59.1% to 77.1% and the share with a physician owner increasing from 11.4% to 29.1%. During this period, markets with only 1 large chain had $495.08 higher commercial prices for outpatient hemodialysis and $564.56 higher medical director compensation per patient compared to markets without large chain facilities.

**Meaning:**

This study highlights the growing consolidation in the US dialysis industry, both horizontally and vertically, and its associations with higher commercial prices for outpatient hemodialysis and compensation for medical directors.

## Teaser

This economic evaluation documents the consolidation of dialysis facilities through chain ownership and vertical integration between dialysis facilities and physicians, as well as studies associations between consolidation and commercial prices for outpatient hemodialysis and medical director compensation.

## Introduction

The dialysis industry has changed substantially over the past 2 decades, with the 2 largest chains, DaVita and Fresenius, now owning nearly 80% of facilities across the US, up from less than 60% in 2005. Amid mounting evidence that consolidation has considerably increased prices at US hospitals,^1^ the high and rising cost of dialysis^2,3^ requires a similar analysis to understand how the market power of these 2 chains affects the 500 000 patients receiving dialysis across the country and the amount they spend on care.^4^

We study the evolving market structure of the dialysis industry and its association with commercial prices for outpatient hemodialysis and medical director compensation. In competitive markets, a patient’s ability to choose among different facilities may restrain prices and promote high-quality care. When a handful of powerful firms dominate the market and inhibit that choice, prices may rise and quality may fall. Similarly, firms with monopsony power may be able to pay their employees less than firms that face more competition.

In health care, market structure has multiple, interrelated dimensions. Horizontal consolidation, where a common owner controls multiple facilities, can empower chains to negotiate higher prices with insurers and reduce salaries for employees. It may also result in lower-quality care, as documented by Erickson et al^5^ and Eliason et al.^6^

The dialysis industry has also evolved along vertical dimensions, such as joint ventures between chains and physicians in which they share ownership of a facility, an arrangement that may entice physicians to steer patients to the facilities they own. Academics, advisory groups, and regulators have all called for scrutiny of the dialysis industry and physician owners, often citing a lack of data as a major hindrance to conducting such research.^6,7,8^ Facilities also employ medical directors to oversee their patients’ care, with unknown influence on where they refer patients for treatment.

In this economic evaluation, we study the association of market structure with commercial prices and medical director compensation in dialysis from 2005 to 2019, considering both horizontal and vertical consolidation. To do so, we use novel data that allow us to track physician medical directors and owners over time.

## Methods

### Data Sources

We combined data from 5 sources. First, we filed a Freedom of Information Act request for physician owners of dialysis facilities from the Medicare Provider Enrollment, Chain, and Ownership System (PECOS). Using these data, complemented by freestanding dialysis facility cost reports compiled by the Centers for Medicare & Medicaid Services (CMS), we identified physician owners of freestanding dialysis facilities from 2005 to 2019 (Lin et al^9^ used a sample of these data that covers only 2017). We provide details of this identification process in the eMethods in Supplement 1. A second Freedom of Information Act request from the ESRD (end-stage renal disease) National Coordinating Center provided data for medical directors of ESRD facilities from 2003 to 2021.

We obtained data on facility characteristics, chain affiliation, and costs, including medical director compensation, from the Healthcare Cost Report Information System maintained by CMS. For data on physician attributes, we used CMS’s National Plan and Provider Enumeration System from 2005 to 2020 and Physician Compare data from 2014 to 2023.

We used data from Medicare claims and facility annual surveys from the United States Renal Data System to identify active facilities and their chain affiliations. We further used the Federal Trade Commission’s Decision and Order files, web searches, and CMS Provider of Service data to document detailed changes of ownership and divestitures of dialysis facilities. See the eMethods in Supplement 1 for more details on facility and ownership identification.

For commercial prices, we used claims data from the Health Care Cost Institute for enrollees in employer-sponsored health plans from 2012 to 2020. We report summary statistics for the prices paid for outpatient hemodialysis claims by hospital service area (HSA) by the number of large dialysis chains present in the HSA. We defined large chains to be the 5 largest over this period (DaVita, Fresenius, US Renal Care, American Renal Associates, and Dialysis Clinic Inc), consistent with the privacy rules in our data use agreement that require at least 5 chains for each reported price. We also compared these prices to Medicare rates.

Finally, we used zip code population data from the 2013 5-year American Community Survey for 2003 to 2013 and the 2021 five-year American Community Survey for 2014 to 2019. Zip code–HSA crosswalks are from Dartmouth Atlas data.

This study was approved by the institutional review board of Duke University with a waiver of informed consent owing to use of deidentified Medicare data. We followed the Strengthening the Reporting of Observational Studies in Epidemiology (STROBE) reporting guidelines for cohort studies.

### Study Population

We studied freestanding dialysis facilities from 2005 to 2019 and physicians who (1) owned a share of a dialysis facility (we began this analysis in 2005 due to concerns about the reliability of PECOS data before then^10^) or (2) directed a facility as indicated in the National Coordinating Center data. Ownership data in PECOS are restricted to individuals and organizations with at least a 5% stake.

Although CMS stipulates that all facilities must have a medical director, many do not report one. Missing medical director information was concentrated in earlier years; the share of facilities without a listed director dropped from 63.7% in 2005 to 11.7% by 2019.

The final facility panel contains 99 496 unique facility-years. Due to different data sources, each result section covers a different segment of this population. eFigure 1 in Supplement 1 provides a flowchart for more details.

### Statistical Analysis

We report the share of freestanding facilities owned by DaVita and Fresenius and the share of the population living in different types of HSAs (eg, with only DaVita or Fresenius facilities) from 2005 to 2019. We also documented how dialysis facility ownership and administration have changed over this period. We further report the monthly mean price per dialysis session in markets with different numbers of large chain facilities and the mean annual compensation for medical directors in markets with different numbers of large chains. We also tested the difference in average price and medical director compensation by the number of chains present in the HSA, as well as the presence of independent or physician-owned facilities, using regressions that controlled for the month of service and the total number of facilities in the HSA. We linearly regressed each of these outcomes on indicator variables for each possible number of chains present in an HSA along with a set of fixed effects for each month by year of the data and for each possible number of facilities present in an HSA. In alternative specifications, we also included indicator variables for whether at least 1 independent facility or at least 1 physician-owned facility was present in the HSA. We assessed statistical significance and calculated 95% CIs using standard errors clustered at the HSA and month-by-year level.

Statistical analyses were performed using Stata/SE, version 18 (StataCorp). Data were analyzed from April 2024 to April 2025.

## Results

### Horizontal Integration

**Figure 1.**
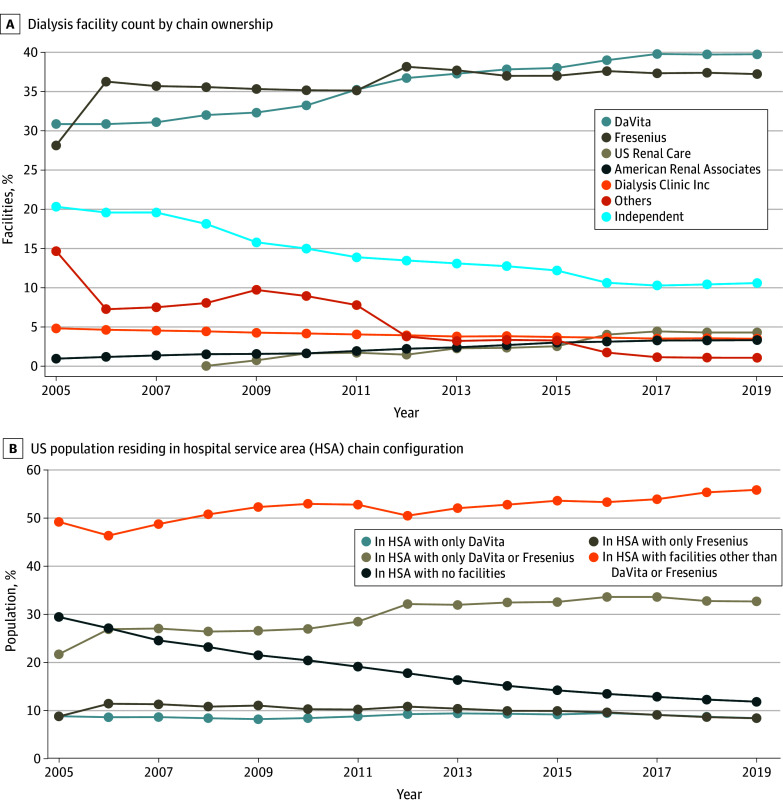
Trends in Market Concentration A, Active facilities were identified using United States Renal Data System facility survey files and patient treatment records. Chain owners were identified using survey files, merger and acquisition records, Federal Trade Commission records, and Centers for Medicare & Medicaid Services Provider of Service data. Further details are provided in the eMethods in Supplement 1. eTable 10 in Supplement 1 shows the corresponding values in the figure. B, Zip code–level population data are from the 2013 five-year American Community Survey for 2003 to 2013 and from the 2021 five-year American Community Survey for 2014 to 2019. Zip code–HSA crosswalks are from Dartmouth Atlas data. eTable 11 in Supplement 1 shows the corresponding values in the figure. eFigure 5 in Supplement 1 shows a longer period from 2000 to 2019. eFigure 3 in Supplement 1 shows the share of DaVita and Fresenius’s new market entries that occurred in HSAs without any existing facility.

Figure 1A shows the share of facilities owned by chains. The combined share of DaVita and Fresenius increased from 59.1% in 2005 to 77.1% in 2019, while the share owned by independent facilities decreased from 20.4% to 10.6%. During this period, several large acquisitions by DaVita and Fresenius contributed to the increase in market concentration. eTable 1 in Supplement 1 contains a list of these acquisitions.

Figure 1B shows the share of the national population residing in an HSA with only facilities owned by DaVita or Fresenius. While the population without any dialysis facility in their HSA decreased from 29.3% in 2005 to 11.7% in 2019, the population that had access to only DaVita or Fresenius facilities increased from 21.6% to 32.5%. Among markets that already had facilities in 2005, the population that had access to only DaVita or Fresenius facilities increased from 30.5% to 34.3%. See eFigure 2 and eTable 2 in Supplement 1 for more detailed statistics.

eFigure 4 in Supplement 1 maps the state-level population shares that had only DaVita or Fresenius facilities in their HSAs. In 2019, Minnesota had the highest share of a DaVita- or Fresenius-only population (99.0%), while Rhode Island, Utah, and Washington, DC, had the lowest (all 0%).

### Vertical Integration

**Figure 2.**
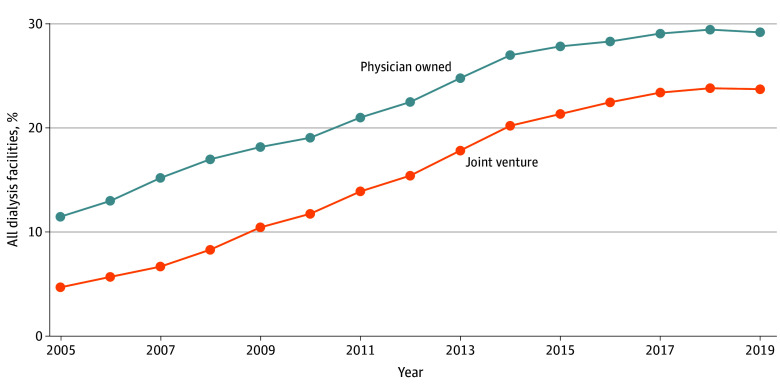
Trends in Dialysis Facility Ownership, 2005-2019 Percentages of all dialysis facilities in the US are presented. Physician-owned facilities include all facilities in which a physician holds an ownership share of 5% or more. These include both individual owners and institutional owners who can be linked to an individual physician. Joint ventures are facilities that simultaneously have a physician owner and a chain owner. For detailed numbers, see eTable 3 in Supplement 1. For detailed definitions and data sources, see the eMethods in Supplement 1.

Figure 2 shows trends in physician ownership. For the baseline analysis, both individual physician owners and physicians linked through institutional owners were included (eMethods in Supplement 1). eTable 3 in Supplement 1 includes a different measure that limits the sample to individual owners, and eTable 4 in Supplement 1 compares these 2 ownership measures for DaVita facilities with the number of physician-owned facilities posted on DaVita’s website.

In the baseline measure, the share of reported physician-owned freestanding facilities increased from 11.4% in 2005 to 29.1% in 2019. A large portion of physician-owned facilities are jointly owned by chains, referred to as joint ventures (JVs). The share of JV facilities increased from 4.6% in 2005 to 23.7% in 2019, respectively comprising 42.1% and 81.1% of physician-owned facilities each year.

eTable 5 in Supplement 1 shows the exclusive nature of physician ownership and medical directorships. The average owner of a DaVita facility has a mean ownership in 2.80 (95% CI, 2.65-2.94) DaVita facilities and virtually no others, while the average owner of a Fresenius facility owns a mean of 4.19 (95% CI, 3.79-4.60) Fresenius facilities and virtually no others. There were similar patterns of exclusivity for medical directors. Chains also paid higher salaries to their medical directors, as shown in eTables 6 and 7 in Supplement 1.

### Integration and Prices

**Figure 3.**
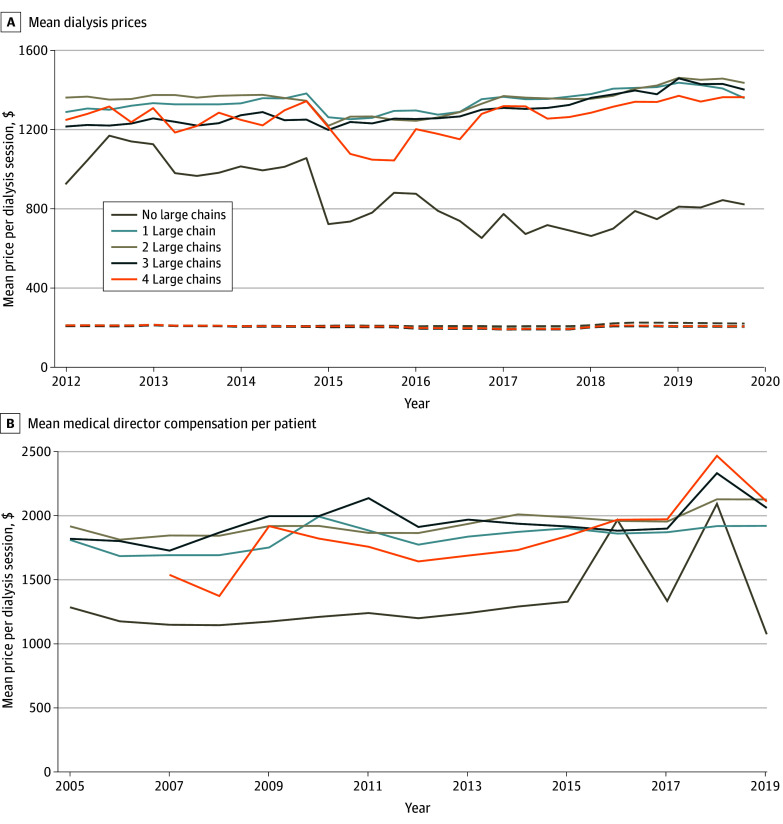
Prices and Compensations per Patient A, Solid lines represent monthly private prices from the Health Care Cost Institute, including all medical claims for enrollees in employer-sponsored health insurance plans offered by carriers. Dashed lines represent monthly Medicare price from United States Renal Data System Medicare claims. B, Solid lines represent yearly per-patient medical director compensation from the Healthcare Cost Report Information System. All dollar amounts are inflation adjusted to 2019 values. Annual compensation is winsorized at the first ($11 356) and 99th ($397 723) percentiles in the pooled sample from years 2005 to 2019. Medicare prices are winsorized at the first ($72) and 99th ($364) percentiles in the pooled sample from years 2005 to 2019. Private prices drop nonpositive allowed amount and the top 1% highest prices. Large chains include DaVita, Fresenius, US Renal Care, American Renal Associates, and Dialysis Clinic Inc.

Figure 3A reports the monthly mean price of outpatient hemodialysis for HSAs with different numbers of large chain facilities. Commercial prices were much higher in markets served by at least 1 large chain compared to markets with only independent facilities and small chains. Markets without a large chain facility had a decline in the private price per session from $929.40 at the beginning of the sample to $827.49 at the end, whereas prices in markets with exactly 1 large chain increased from $1292.91 to $1362.76. Commercial prices did not differ by the number of chains operating in the market. There were similar differences in per-patient medical director compensation (Figure 3B).

The Table compares the private price for dialysis across markets, adjusting for changes in price over time and differences in the total number of facilities in the market. Markets without any large chain facilities had much lower prices than the reference group of markets with only a single large chain. Compared to these large chain monopoly markets, prices in markets without large chain facilities were $495.08 lower (95% CI, −$619.85 to −$371.21), or 37.1% less than the mean price in monopoly markets of $1333.90 (95% CI, $1282.74-$1385.13). However, prices did not differ much when more than 1 large chain operated in the HSA, as markets with 2, 3, and 4 large chains had no meaningful difference in price relative to monopoly markets. HSAs with at least 1 independent facility had mean prices $112.48 lower (95% CI, −$160.97 to −$63.99), or 8.0% less, than the mean price in markets without independent facilities of $1409.80 (95% CI, $1352.55-$1467.04), while HSAs with at least 1 JV had mean prices that were $29.18 higher on average (95% CI, −$14.53 to $72.88). Taken together, these results show that markets with independent facilities had much lower mean prices than those with just large chains.

**Table.** Private Price for Hemodialysis and Medical Director Per-Patient Compensation Across Market Types

Facility characteristic	Coefficient (95% CI)					
	Private claim price, $ per single dialysis session^a^			Medical director per-patient compensation, $^b^		
	Model 1	Model 2	Model 3	Model 4	Model 5	Model 6
In HSA with no top 5 chain^c^	−495.08 (−618.95 to −371.21)	−399.55 (−531.33 to −267.78)	−395.77 (−527.62 to −263.92)	−564.56 (−700.10 to −429.03)	−481.44. (−617.48 to −345.39)	−486.94 (−623.54 to −350.34)
In HSA with 1 top 5 chain^d^	0 [Reference]	0 [Reference]	0 [Reference]	0 [Reference]	0 [Reference]	0 [Reference]
In HSA with 2 top 5 chains	31.42 (−25.51 to 88.36)	5.38 (−51.22 to 61.99)	−0.49 (−56.50 to 55.51)	244.15 (126.28 to 362.02)	212.76 (87.43 to 338.08)	237.17 (107.19 to 367.16)
In HSA with 3 top 5 chains	−39.85 (−121.61 to 41.92)	−75.29 (−154.56 to 3.99)	−85.57 (−165.46 to −5.69)	332.54 (132.48 to 532.61)	297.09 (87.48 to 506.71)	334.27 (119.90 to 548.63)
In HSA with 4 top 5 chains	−35.23 (−153.41 to 82.96)	−69.80 (−180.02 to 40.42)	−80.99 (−190.16 to 28.19)	281.85 (−14.38 to 578.08)	240.04 (−66.27 to 546.35)	292.00 (−22.64 to 606.63)
In HSA with independent facility	NA	−112.48 (−160.97 to −63.99)	−111.08 (−159.71 to −62.45)	NA	−159.06 (−267.08 to −51.04)	−154.11 (−260.31 to −47.90)
In HSA with joint ventures	NA	NA	29.18 (−14.53 to 72.88)	NA	NA	−154.32 (−248.34 to −60.31)
Constant	1333.90 (1282.74 to 1385.13)	1409.80 (1352.55 to 1467.04)	1395.69 (1333.43 to 1457.96)	1755.12 (1657.89 to 1852.36)	1831.25 (1705.27 to 1957.24)	1886.16 (1755.91 to 2016.41)
No. of observations	2 098 971	2 098 971	2 098 971	69 992	69 992	69 992
Adjusted *R*^2^	0.04	0.04	0.04	0.01	0.01	0.01
Fixed effects on year/month^e^	Yes	Yes	Yes	Yes	Yes	Yes
Fixed effects on No. of facilities in HSA^e^	Yes	Yes	Yes	Yes	Yes	Yes

Abbreviations: HSA, hospital service area; NA, not applicable.

^a^ Monthly private claim price from the Health Care Cost Institute from January 2012 to December 2020. Each observation is a single-session dialysis claim.

^b^ Yearly per-patient medical director compensation for freestanding dialysis centers from the Healthcare Cost Report Information System from 2005 to 2019. Each observation is a facility-year. All dollar amounts are inflation adjusted to 2019 values. Annual compensation is winsorized at the first ($11 356) and 99th ($397 723) percentiles in the pooled sample from years 2005 to 2019.

^c^ The top 5 chains are DaVita, Fresenius, US Renal Care, American Renal Associates, and Dialysis Clinic Inc.

^d^ Samples in markets with 1 top 5 chain as the baseline group were used, meaning coefficients on other market types reflect the mean difference between samples in that market type and samples in markets with 1 top 5 chain facility.

^e^ Fixed effects absorb all variation in the relevant categorical variable (ie, adding 1 dummy variable for each value of the variable). Year/month fixed effects include 1 dummy variable for each month by year; number of facilities in HSA fixed effects include 1 dummy variable for each possible number of facilities present in an HSA. Year/month fixed effects flexibly control for time trends shared by all samples; number of facilities in HSA fixed effects flexibly control for differences in the outcome variable that are purely driven by the number of facilities in the market (eg, via competition). eTable 12 in Supplement 1 compares the number of facilities across market types.

The equivalent Medicare payments for dialysis across markets is summarized in eTable 8 in Supplement 1, with no meaningful differences. Because Medicare adjusts payments to account for differences in operating costs across markets, such as local wage rates, this suggests that chains’ disproportionate entry into high-cost markets explains most of the small difference observed for Medicare rates.

Equivalent regressions for medical director compensation per patient are estimated in the Table. Markets without any large chain facilities had $564.56 lower (95% CI, −$700.10 to −429.03) annual compensation than markets with only 1 large chain. Compensation in markets with independent facilities was $159.06 lower (95% CI, −$267.08 to −$51.04) than those without. There were also associations between having more large chains in a market and higher per-patient compensation for medical directors. Markets with at least 1 JV facility paid $154.32 less (95% CI, −$248.34 to −$60.31) than those without any JV.

## Discussion

The rise of for-profit dialysis chains began shortly after the expansion of Medicare coverage to most US patients receiving dialysis in 1972.^11,12^ National Medical Care, for instance, was already acquiring independent facilities and forming vertical alliances with nephrologists.^13^ This study shows that the trends in consolidation and vertical integration have continued over the past 20 years.

Academics, advisory groups, and regulators have all called for an analysis of the growing consolidation in the dialysis industry and the influence of physician medical directors and owners, often citing a lack of data as a major impediment to conducting such research.^6,7,8^ A letter from the Medicare Payment Advisory Commission to CMS, for example, states that there are “no publicly available, searchable data sources to identify physician ownership interests in a dialysis facility, including…the total number of joint ventures that exist,” so “researchers are not able to examine the effect of joint venture relationships on beneficiaries’ quality of care, Medicare spending, and beneficiaries’ cost sharing.”^14^ Further highlighting the need for greater transparency are a number of recent whistleblower lawsuits that allege vertical ties in dialysis may distort patient care and violate federal laws,^15,16^ such as the Anti-Kickback Statute, which prohibits remuneration for patient referrals.^17^ Another law aimed at mitigating financial conflicts of interest, the Stark Law,^18^ explicitly prohibits physicians from referring patients to entities in which they hold a financial stake, but freestanding dialysis facilities receive an exemption. Much of the consolidation in dialysis has not been subject to antitrust review.^19^

The high commercial prices commanded by DaVita and Fresenius have also come under recent scrutiny. In the 2022 case *Marietta Memorial Hospital Employee Health Benefit Plan v DaVita Inc*, the Supreme Court ruled that the health plan did not violate the Medicare Secondary Payer Act by restructuring its dialysis benefit to avoid paying commercial rates far exceeding Medicare’s.^20^ Similarly, in 2018, California voters rejected ballot initiative Proposition 8, known as the Fair Pricing for Dialysis Act, which would have capped dialysis organizations’ profits at 15% above what they spend on “direct patient care services costs.”^21^

Markets with a high concentration of large chains have much higher commercial prices for dialysis. The limited choices for patients and payers in these markets, even when set against the highly concentrated insurance industry, allows chains such as DaVita and Fresenius to command the highest markups over Medicare of all health care sectors.^22^ Although Erickson et al^5^ found that “a decade of consolidation in the United States dialysis industry did not (on average) limit patient choice or result in more concentrated local markets,” the situation looks much different today: nearly one-third of the US population now lives in an area with only DaVita or Fresenius available to them for dialysis. Previous work suggests that high commercial rates are not due to higher-quality care, as independent facilities’ quality decreased after being acquired by a large chain.^23^ In eTable 9 in Supplement 1, we constructed a facility-level standardized 1-year mortality rate and found no difference across market types.

Large chains do not appear to use their monopsony power over medical directors to pay lower salaries, an approach seemingly at odds with a conventional corporate strategy of maximizing profits through minimizing labor costs. Instead, we found that chains pay their medical directors much higher salaries than independent facilities do. When chains face more competition in a market, their medical directors receive higher per-patient pay.

The growing use of joint ventures in dialysis may further entrench the large chains and foreclose entry by rivals.^15,16,24^ For the Anti-Kickback Statute and Stark Law to work as intended, regulators must first have access to data that allow them to link referrals to ownership shares and medical director compensation. To our knowledge, this work is the first to move them closer to doing so.

### Limitations

This study faces 2 main limitations. First, there may be measurement error or missing entries in the administrative data. Although facilities must report ownership data to CMS, form-response errors or inconsistencies among facilities may lead to errors in how physician ownership is coded. Moreover, medical director responsibilities may be shared by several physicians within a group practice, but the facility may designate only 1 as the listed medical director in the present data. Second, we manually matched a small number of owners in PECOS by their names or addresses, a process that could be subject to replication issues based on the judgment of the replicator. See eMethods in Supplement 1 for a detailed discussion.

## Conclusions

Results of this economic evaluation support that over the past 2 decades, the dialysis industry has become the most concentrated of all health care sectors.^25^ In line with this consolidation, dialysis has the highest markup over Medicare for commercial insurers.^22,26^ More recently, the largest dialysis chains have enhanced their financial ties with physicians by offering ownership stakes in facilities and paying substantially higher salaries to serve as medical directors.

Attempts to study ownership ties in the dialysis industry have been hindered by a lack of data. By updating facility ownership data and compiling the first panel of owners and directors in the literature, we document several novel and important facts: (1) physicians’ ownership stakes have become much more common in dialysis, (2) compensation for medical directors varies widely across large chains and independent facilities, (3) a substantial portion of the US population now has access to only DaVita or Fresenius, and (4) commercial prices are much higher in areas with only large chains. With better access to data on the identities of medical directors and owners of dialysis facilities, regulators and patient advocates will have a better understanding of how care might be influenced by these relationships.

## Notes

Supplement 1.
eMethods
**eTable 1.** List of Main Merger & Acquisition Events **eTable 2.** National Population Shares by HSA Types among Existing Markets in 2005, from 2005 to 2019 **eTable 3. **Time Trends in Medical Director and Physician Ownership Characteristics from 2005 to 2019 **eTable 4. **DaVita Physician-owned Facility Validation **eTable 5. **Distribution of Chains Owned/Directed Among Owners/Medical Directors, 2019 **eTable 6.** Freestanding Dialysis Facility Medical Director Compensation Summary Statistics by Chain, 2005 and 2019 **eTable 7. **Freestanding Dialysis Facility Medical Director Compensation per Patient Summary Statistics by Chain, 2005 and 2019 **eTable 8. **Effects on Medicare Price for Hemodialysis across Markets, OLS regressions **eTable 9. **Effects on Standardized 1-Year Mortality Rate (SMR), OLS regressions **eTable 10. **Facility Count Shares by Chains from 2005 to 2019 **eTable 11. **National Population Shares by HSA Types from 2005 to 2019 **eTable 12. **Comparison of Facility Counts across Market Types **eFigure 1. **Study Flowchart **eFigure 2. **Share of National Population by HSA Types among Existing Markets in 2025, 2005 to 2019 **eFigure 3. **Share of Entries into Markets with No Existing Facilities **eFigure 4. **State-level Population Shares in HSAs with only DaVita or Fresenius **eFigure 5. **Trends of Market Concentration
eReferences

Supplement 2.
Data Sharing Statement
