# Clarification of Conflict of Interest Disclosures

**DOI:** 10.1001/jamahealthforum.2025.5593

**Published:** 2025-11-07

The Original Investigation titled “Financial Ties, Market Structure, Commercial Prices, and Medical Director Compensation in Dialysis,”^1^ published online June 18, 2025, has been updated to provide more detail in the Conflict of Interest Disclosures section. Ryan C. McDevitt, PhD, has served as a paid consultant and litigation expert for Charles River Associates on matters in the health care sector, including cases where he supported corporations being sued by providers of kidney dialysis services, such as DaVita and Fresenius, that were evaluated in the study. This article was corrected online.

## References

[1] Xia X, Deng W, Eliason PJ, . Financial ties, market structure, commercial prices, and medical director compensation in dialysis. JAMA Health Forum. 2025;6(6):e252659. doi:10.1001/jamahealthforum.2025.2659 40531510 PMC12177639

