# Supplementary material for: Financial Ties, Market Structure, Commercial Prices, and Medical Director Compensation in Dialysis
Source: JAMA Health Forum. 2025 Jun 18;6(6):e252659. doi: 10.1001/jamahealthforum.2025.2659 (PMC12177639; doi:10.1001/jamahealthforum.2025.2659)
Supplement: Supplement 1. — eMethods eTable 1. List of Main Merger & Acquisition Events eTable 2. National Population Shares by HSA Types among Existing Markets in 2005, from 2005 to 2019 eTable 3. Time Trends in Medical Director and Physician Ownership Characteristics from 2005 to 2019 eTable 4. DaVita Physician-owned Facility Validation eTable 5. Distribution of Chains Owned/Directed Among Owners/Medical Directors, 2019 eTable 6. Freestanding Dialysis Facility Medical Director Compensation Summary Statistics by Chain, 2005 and 2019 eTable 7. Freestanding Dialysis Facility Medical Director Compensation per Patient Summary Statistics by Chain, 2005 and 2019 eTable 8. Effects on Medicare Price for Hemodialysis across Markets, OLS regressions eTable 9. Effects on Standardized 1-Year Mortality Rate (SMR), OLS regressions eTable 10. Facility Count Shares by Chains from 2005 to 2019 eTable 11. National Population Shares by HSA Types from 2005 to 2019 eTable 12. Comparison of Facility Counts across Market Types eFigure 1. Study Flowchart eFigure 2. Share of National Population by HSA Types among Existing Markets in 2025, 2005 to 2019 eFigure 3. Share of Entries into Markets with No Existing Facilities eFigure 4. State-level Population Shares in HSAs with only DaVita or Fresenius eFigure 5. Trends of Market Concentration eReferences [file jamahealthforum-e252659-s001.pdf]

Supplemental Online Content

Xia X, Deng W, Eliason PJ, et al. Financial ties, market structure, commercial prices, and medical director compensation in dialysis. *JAMA Health Forum*. Published online June 18, 2025. doi:10.1001/jamahealthforum.2025.2659

eMethods
eTable 1. List of Main Merger & Acquisition Events
eTable 2. National Population Shares by HSATypes among Existing Markets in 2005, from 2005 to 2019
eTable 3. Time Trends in Medical Director and Physician Ownership Characteristics from 2005 to 2019
eTable 4. DaVita Physician-owned Facility Validation
eTable 5. Distribution of Chains Owned/Directed Among Owners/Medical Directors, 2019
eTable 6. Freestanding Dialysis Facility Medical Director Compensation Summary Statistics by Chain, 2005 and 2019
eTable 7. Freestanding Dialysis Facility Medical Director Compensation per Patient Summary Statistics by Chain, 2005 and 2019
eTable 8. Effects on Medicare Price for Hemodialysis across Markets, OLS regressions
eTable 9. Effects on Standardized 1-Year Mortality Rate (SMR), OLS regressions
eTable 10. Facility Count Shares by Chains from 2005 to 2019
eTable 11. National Population Shares by HSA Types from 2005 to 2019
eTable 12. Comparison of Facility Counts across Market Types
eFigure 1. Study Flowchart
eFigure 2. Share of National Population by HSA Types among Existing Markets in 2025, 2005 to 2019
eFigure 3. Share of Entries into Markets with No Existing Facilities
eFigure 4. State-level Population Shares in HSAs with only DaVita or Fresenius
eFigure 5. Trends of Market Concentration
eReferences

This supplemental material has been provided by the authors to give readers additional information about their work.

© 2025 Xia X et al. *JAMA Health Forum*.

eMethods

A.1 Identifying facilities and chain ownership

To identify active facilities each year, we leverage both the CMS annual survey data and patient treatment records from USRDS. We require the facility-year to either show up in the survey data, or have more than one patient with center hemodialysis, center self hemodialysis, home hemodialysis, hemodialysis training, CAPD, CAPD training, or other peritoneal dialysis treatments supervised by the facility. We also require the year to be between the CMS certification and termination years if this information is available.

The chain ownership variable from USRDS may not update immediately after changes of ownership, so we clean the chain ownership variables using additional information from M&A records, FTC records, CMS Provider of Service (POS) data, etc. For mergers with divestiture orders from the FTC, we track down each divestiture facility if possible, and fix their chain ownership and the date of the change. POS data provides the dates for the change of ownership, which we use to pinpoint the exact date of the chain ownership transition and fill in missing values. eTable 1 provides a list of M&As that we checked and fixed in the data.

A.2 Identifying physician owners

To identify which facilities have physician owners as well as the identity of those physicians, we use three sources of data: Historical records of the Medicare Provider Enrollment, Chain, and Ownership System (PECOS) on dialysis facilities and physician groups (we refer to them as ESRD PECOS and PG PECOS respectively hereafter) --- both were received through Freedom of Information Act (FOIA) requests --- and the complete archive of registry records of all companies in North America from OpenCorporates. In the two PECOS datasets, the start of the earliest association goes back to 1800, and the latest dissociation goes beyond June 2024, with many associations still ongoing. The OpenCorporates data contains the most recent record from all jurisdictions in the US, currently active or not, up until February 2025, with the earliest incorporation date going back to 1800.

The Medicare Provider Enrollment, Chain, and Ownership System (PECOS) is an online enrollment management system that allows care providers to enroll as Medicare providers or suppliers. The ESRD PECOS comes from the application form CMS-855A, which is for institutional providers; the PG PECOS comes from CMS-855B, which is filled out by clinics, group practices, and certain other suppliers. For simplicity, in the passage below, we refer to entities in PG PECOS only as "physician groups." Both forms are required by the corresponding entities in

order to enroll in the Medicare program and receive a Medicare billing number. A new form must also be submitted when an entity experiences changes in enrollment information (e.g. changes of ownership, managing employees,...).

PECOS distinguishes between individual owners and organizational owners. In ESRD PECOS, we first identify all group and individual owners of dialysis facilities. The ownership file provides a list of facility owners, managers, directors, etc., each with a unique owner ID number, linked to a facility enrollment number, stop and start dates of role tenure, owner role description, ownership percentage, and owner name. To be considered an "owner" in the PECOS data, the role description of that entity has to contain the word "owner", "sole proprietor", or "partnership", which include these role descriptions: "5% or greater direct ownership interest", "5% or greater indirect ownership interest", "General partnership interest", "Limited partnership interest", "Sole Owner", and "Sole proprietor". Entities with role descriptions such as "Operational/managerial control" and "director" were not considered owners.

If an individual owner has a National Provider Identifier (NPI), it is listed in the data, which we later merge with other data sources to identify physicians. We conducted detailed sanity checks on the NPI-individual owner linkage and confirmed its accuracy.

However, many joint ventures are formed between dialysis chains and physician group practices or physician LLCs, so these ownerships may be recorded at the company level instead of individual physicians. To identify these types of physician ownership, we link organization owners in PECOS to individual physicians via additional matching procedures. ESRD PECOS lists the name and PECOS Associate Control ID (PAC ID) of each organization owner, and includes various types of organizations: Institutional investors, hospital systems, physician groups, etc. For the purpose of identifying physician ownership, we focus on organization owners that can be linked to local physician groups, physician LLCs, or clinics. To do so, we develop a procedure to link organization owners in ESRD PECOS to entities listed in Physician Group PECOS (PG PECOS). Meanwhile, we require the entity linked in PG PECOS to have at least one individual physician owner to ensure that at least one physician has an ownership connection to the dialysis facility. PG PECOS has slightly different owner role descriptions from ESRD PECOS, and we include individual physicians as owners of the physician group if and only if they have any of these roles: "5% or more ownership interest", "Partner", "Sole owner", or "Sole Proprietor."

When linking organization owners in ESRD PECOS to entities in PG PECOS, we first perform a direct name match, which matches 497 unique organization names. Other potential owners that are not matched mainly due to two issues: first, their names in the ESRD PECOS as an organization owner is different from the name listed in PG PECOS,

© 2025 Xia X et al. *JAMA Health Forum*.

for example, one is a letter abbreviation and the other is in full spelling; or second, which is more common, is that the ownership of the dialysis facility is registered at a financial entity that is different from the operating entity registered in PG PECOS. For example, the facility ownership can be registered using the physician's trust, while PG PECOS only lists the corresponding physician LLC; or the physicians in a group practice have another partnership/investment entity, which they use to register the ownership of the dialysis facility. We found numerous such cases when examining JVs listed in the antitrust lawsuit (Barbetta vs. DaVita) and FTC divestitures.

To overcome these and link related entities from ESRD PECOS and PG PECOS, we further leverage data from OpenCorporates. To our best knowledge, OpenCorporates is one of the world's largest open databases on legal entities, including corporations, LLCs, partnerships, nonprofits, etc., collected from company registries and other public records. OpenCorporates provided us with the complete archive of all jurisdictions in the US, which includes the most recent record of all companies that ever showed up in their data. We use these data to first match organization owners in ESRD PECOS to OpenCorporates, by business name, and find the individual agents listed under that company. Among all 3,107 unique organization owner names to be matched, in this step, we managed to link 2,812 (91%) of them on OpenCorporates and find at least one agent listed. Among the rest, most are from New Jersey. OpenCorporates doesn't have company officer information for New Jersey. Therefore, we hand-searched all organization owners in New Jersey on other sites, mainly Bizapedia, to fill in the missing company agent names. All together, we were able to match more than 94% of the organization owner names to at least one agent listed under the entity.

We then match the linked agents to members of physician groups listed in PG PECOS. We allow the agents to be matched with any member of the physician group, not just owners as defined above, since they are still informative in implying the two entities are related. Given a positive match between a facility's owning entity's agent and a physician, we classified that facility as having a physician owner. A common issue of matching people's names is shared/common names. To eliminate false matching as much as we can, we impose two more restrictions. First, we require the linked physician group to be in the same state as the dialysis facility that the organization holds ownership of, which means that the agent names we use to connect must be from the state the dialysis facility is located; second, we drop agent names that are matched to physician groups in multiple states, since it is very likely the name is relatively generic and commonly shared. After this, we map the physician owners of the linked physician group to the dialysis facility.

© 2025 Xia X et al. *JAMA Health Forum*.

In the end, since our focus is on identifying local physician owners of dialysis facilities, we go through all the organization owners that are linked to a physician group with an owner who has an NPI, and drop any remaining institutional investors, hospitals, medical centers, universities, or healthcare systems. We are left with 1,277 unique organization owners that are linked to an individual owner with an NPI, which are fed to the next step for identifying JVs.

After identifying individual owners with NPIs of dialysis facilities, we merge that information to create an owner-facility-month panel. Both ESRD PECOS and PG PECOS detail the specific date of ownership changes. So, if an individual owner is linked to the facility via a physician group, for each *dialysis facility–individual owner* pair, we assume the individual holds ownership at the facility during the overlapped period of: the organization owner's ownership tenure of the dialysis facility, and, the individual's ownership tenure of the physician group.

In this process, we only keep *physician* owners, excluding NPIs corresponding to nurse practitioners, physician assistants, etc. We define physicians by the "physician specialties" CMS uses for the Health Professional Shortage Area (HPSA) Bonus Program, which generally includes physicians "who are M.Ds, D.Os, D.Cs, D.P.Ms, and O.Ds."[1]

A facility is defined as physician-owned in a month/year if and only if there is at least one physician owner identified in that month/year. A dialysis facility is a JV if it's physician-owned and it's owned by a dialysis chain. Moreover, as mentioned in the next section, since all dialysis facilities under the chain American Renal Associates (ARA) are known to have physician owners, we treat all ARA facilities as physician-owned and JV in our analysis.

A.3 Validation process

As no comprehensive data on the prevalence of dialysis physician ownership exists, we found four external sources providing partial pictures of physician ownership prevalence, and compared our dataset against those. These sources are:

1) American Renal Associates:

   American Renal Associates (ARA) is a unique dialysis chain in that all of the facilities are joint ventures. Under our current methodology, we identify 88.1% of ARA facility-years as being a joint venture. As mentioned above, in our analysis, we treat all ARA facilities as physician-owned and JV in our analysis.

2) Joint venture counts from DaVita's Website

On DaVita's website, they display the number of joint ventures that they operate over the years 2008 to 2018[2]. We find that over the years, our estimates are similar to their counts, with the similarity improving over time as seen in eTable 4. One complication of this comparison is that it is unclear what DaVita is counting as a joint venture facility. We reached out to DaVita to seek clarification, but received no response. In eTable 4, we compare two different measures of joint ventures, one that includes all identified owners (baseline), and the other that only includes individual owners as shown in the ESRD PECOS, dropping physicians linked via organization owners. This comparison shows the value of linking organization owners with physicians: if we only consider individual physician owners as listed in ESRD PECOS, we'd miss many JVs that even DaVita themselves reported. Parallelly, we also find that Fresenius has very few physician owners directly listed as individual owners in ESRD PECOS, yet organization owners uncover many of them.

3) Whistleblower case against DaVita:

   DaVita was hit with a whistleblower lawsuit (Barbetta vs. DaVita) alleging patient steering and kickbacks among dialysis facilities that were joint ventures in 2014. The resulting court documents (a corporate integrity agreement and the lawsuit documentation) provide us with a list of 49 validated joint venture facilities. Of these facilities, we correctly identify 36/49 as being joint ventures. The unidentified owners do not have PECOS individual or group owners that would qualify them to be joint ventures under any definition (Blackrock, Davita Inc., etc.).

4) Fresenius FTC Filing:

   In 2012, the Federal Trade Commission investigated Fresenius and ordered it to divest from 30 dialysis facilities. The resulting court documents provide us with a list of 30 validated joint venture facilities. With our methodology, we correctly identify as joint ventures 22/30 of those.

A.4 Potential Bias in Ownership Identification

As mentioned above, there is no comprehensive data on the prevalence of dialysis physician ownership at this point. Our goal is to leverage the best data sources available and develop a procedure as systematic and coherent as we can. Due to the overall data quality, however, there is still room for bias in our measure:

1. Data reporting errors. The quality of PECOS data can improve over time, yet it is still subject to reporting errors. It's believed that by 2005, PECOS data would include the updated records for most providers,[3] which is why we focus on years since 2005.
2. We regard our measure as relatively conservative. For organization owners, we only focus on those that can be matched to a physician group in the same state where the dialysis facility locates, via agents we linked on OpenCorporates; we dropped organization owners that are registered in multiple states, and; we exclude physicians that serve as directors, officers, etc. for the dialysis facility or physician group. As a result, the ownership we identified is more direct and evident in our data. Physicians could certainly have ownership of dialysis facilities through more obscure channels, such as via shell companies, investment companies, or even family members and friends. We didn't find any systematic ways to identify these types of ownership, and it is beyond the scope of this study. Moreover, we exclude ownerships through large hospital and healthcare networks, or through multi-state physician groups, since we want to focus on the physician ownership that can be linked to individual physicians as much as we can. We also didn't find a systematic way to include them: many dialysis facilities could just be a subsidiary of a (university) hospital network, so the ownership by the hospital network doesn't necessarily reflect the type of physician ownership we are interested in for this study.
3. Potential mismatch. As mentioned above, during the matching process, we sometimes have to match company or agent names manually, due to the unstandardized data structure, which may introduce errors. To minimize these errors, as mentioned above, we put detailed conditions on those matchings (e.g., requiring agent names to be in the same state and dropping names matched in multiple states), and had multiple members from the team to validate the matchings. The individual cases from the lawsuits and divestitures also provide great guidance on potential sources of bias and errors, and allow us to learn different linkage structures, embed them into our procedure, while keeping the entire workflow internally consistent and suitable for our study purpose.

A.5 Identifying medical directors

To identify medical directors, we employ a list provided by the ESRD NCC in response to our FOIA request. This list detailed the first and last name of each medical director, the facility they directed, and the dates over which they performed that service. We matched the first name and last name of the medical director for each

given facility-month to NPPES. Some medical directors did not match (reasons for this include that their medical license may have expired and transcription errors). In these cases, we performed a manual search for them in Doximity, Healthgrades, or other directories of physicians.

A.6 Analysis Sample in the Health Care Cost Institute (HCCI) Data

In the HCCI data, we obtain samples from 2012 to 2020 at the claim level. Following previous work[4], for each patient, we use her single session dialysis claims in the first 33 months of dialysis. We limit samples to patient-months with fewer than 15 treatments. We also drop claims with non-positive allowed amounts or with the top 1% highest dialysis prices.

© 2025 Xia X et al. *JAMA Health Forum*.

eTable 1: List of Main Merger & Acquisition Events

Date	Acquiror	Target	Target Facilities	Divestiture facilities
2001 Jan	Fresenius	Everest	74	NA
2004 Apr	RCG	National Nephrology Association	87	NA
2005 Oct	DaVita	Gambro	565	Mandate divestiture of 68 clinics to Renal Advantage and one clinic to its medical directors and their partners, and end two management services agreements through which it manages clinics on behalf of third-party owners
2006 Mar	Fresenius	RCG	450	Mandate divestiture of 91 clinics and RCG's joint venture equity interests in 12 additional clinics to NRI/DSI
2009 Jan	Renal Advantage	National Renal Alliance	45	NA
2010 Jun	USRC	Dialysis Corporation of America	84	NA
2011 Jan	Liberty	Renal Advantage	106	Mandate divestiture of 29 clinics to Newco
2011 Sep	DaVita	DSI	112	NA
2012 Mar	Fresenius	Liberty/ Renal Advantage	260	Mandate divestiture of 60 clinics to Newco
2013 Aug	USRC	Ambulatory Services of America/IDS	79	NA
2016 Mar	USRC	DSI	99	Mandate divestiture of 3 clinics in Laredo to Satellite Healthcare, Inc.
2017 Mar	DaVita	Renal Ventures	38	Mandate divestiture of 7 clinics to PDA-GMF Holdco, LLC, a joint venture between Physicians Dialysis and GMF Capital LLC

© 2025 Xia X et al. *JAMA Health Forum*.

eTable 2: National Population Shares by HSATypes among Existing Markets in 2005, from 2005 to 2019 (%)[a]

Year	With only DaVita	With only Fresenius	With only DaVita or Fresenius	With Facilities Other than DaVita or Fresenius	With Any Facility	With No Facility
2005	12.28	12.19	30.51	69.49	100.00	0.00
2006	11.46	15.16	36.33	63.38	99.71	0.29
2007	10.90	14.42	35.06	64.41	99.47	0.53
2008	10.34	13.36	33.30	66.12	99.41	0.59
2009	9.70	13.18	32.45	66.81	99.25	0.75
2010	9.68	11.93	32.31	66.64	98.95	1.05
2011	9.83	11.54	33.48	65.52	99.00	1.00
2012	9.90	11.71	36.51	62.44	98.95	1.05
2013	9.74	11.13	35.84	63.29	99.13	0.87
2014	9.51	10.39	35.71	63.38	99.08	0.92
2015	9.14	10.13	35.20	63.78	98.98	1.02
2016	9.33	9.55	36.02	62.93	98.95	1.05
2017	8.69	8.89	35.69	63.26	98.95	1.05
2018	8.29	8.29	34.54	64.52	99.06	0.94
2019	7.97	7.95	34.31	64.81	99.12	0.88

a Share of the national population living in HSAs with different types of facility chain configuration and with existing facilities in 2005.

© 2025 Xia X et al. *JAMA Health Forum*.

eTable 3: Time Trends in Medical Director and Physician Ownership Characteristics from 2005 to 2019[a]

Year	Total Facilities	Percent of Chain Facilities	Percent of Physician-owned Facilities, All Owners	Percent of Physician-owned Facilities, Individual Owners Only	Percent of JV Facilities, All Owners	Percent of JV Facilities, Individual Owners Only	Percent of Independent Physician-Owned Facilities, All Owners	Percent of Independent Physician-Owned Facilities, Individual Owners Only	Mean (%95 CI) Med Dir Compensation	Mean (%95 CI) Med Dir Compensation per Patient	Median (%95 CI) Med Dir Compensation	Median (%95 CI) Med Dir Compensation per Patient
2005	4,058	79.65	11.41	10.00	4.63	3.60	6.78	6.40	120,829.83 (117,684.98 to 123,974.68)	1,797.08 (1,721.39 to 1,872.77)	94,251.43 (91,628.09 to 97,962.54)	1,540.06 (1,507.94 to 1,567.01)
2006	4,240	80.38	12.92	11.23	5.64	4.32	7.28	6.91	117,921.20 (114,908.38 to 120,934.01)	1,721.18 (1,683.25 to 1,759.11)	90,182.49 (88,573.19 to 93,729.76)	1,484.94 (1,452.95 to 1,525.64)
2007	4,443	80.37	15.12	13.19	6.62	5.20	8.50	7.99	117,454.11 (114,515.52 to 120,392.70)	1,728.12 (1,690.82 to 1,765.43)	90,877.33 (88,495.30 to 92,591.14)	1,487.10 (1,462.45 to 1,520.65)
2008	4,680	81.84	16.92	14.55	8.25	6.32	8.67	8.23	116,400.26 (113,679.85 to 119,120.67)	1,740.79 (1,703.21 to 1,778.37)	91,319.41 (88,995.79 to 93,791.81)	1,496.39 (1,473.19 to 1,519.98)
2009	4,902	84.17	18.09	15.54	10.38	8.12	7.71	7.42	117,464.20 (114,859.73 to 120,068.66)	1,820.42 (1,772.00 to 1,868.83)	94,448.07 (91,284.18 to 96,090.97)	1,545.76 (1,521.82 to 1,565.71)
2010	5,072	84.98	18.99	16.15	11.69	9.15	7.30	7.00	119,647.86 (117,122.86 to 122,172.85)	1,935.95 (1,805.33 to 2,066.56)	97,786.55 (94,485.10 to 99,657.17)	1,594.96 (1,567.39 to 1,617.96)
2011	5,228	86.07	20.93	17.39	13.83	10.60	7.10	6.79	118,025.72 (115,660.36 to 120,391.07)	1,892.79 (1,817.67 to 1,967.91)	97,123.16 (95,311.77 to 99,609.01)	1,559.41 (1,532.33 to 1,582.21)
2012	5,420	86.49	22.42	18.62	15.35	11.86	7.07	6.76	116,888.42 (114,696.27 to 119,080.56)	1,822.05 (1,784.22 to 1,859.88)	98,388.21 (96,313.12 to 99,793.93)	1,559.87 (1,535.67 to 1,585.12)
2013	5,643	86.87	24.70	19.60	17.76	13.08	6.94	6.52	117,801.74 (115,667.34 to 119,936.15)	1,889.57 (1,845.46 to 1,933.68)	98,769.86 (97,268.55 to 99,801.69)	1,577.89 (1,545.18 to 1,605.25)
2014	5,925	87.22	26.90	20.59	20.14	14.26	6.76	6.33	118,821.36 (116,808.32 to 120,834.41)	1,929.78 (1,837.00 to 2,022.56)	100,310.41 (98,144.33 to 102,592.83)	1,594.44 (1,567.23 to 1,622.28)
2015	6,115	87.77	27.75	20.85	21.26	14.82	6.49	6.03	119,560.56 (117,579.30 to 121,541.83)	1,932.34 (1,892.47 to 1,972.21)	102,075.33 (99,235.26 to 103,518.26)	1,611.47 (1,586.35 to 1,634.37)
2016	6,413	89.33	28.22	20.99	22.39	15.62	5.83	5.37	117,820.16 (115,917.00 to 119,723.31)	1,915.50 (1,872.01 to 1,958.98)	100,260.42 (97,998.99 to 101,679.20)	1,580.44 (1,559.50 to 1,609.36)
2017	6,742	89.69	28.98	21.23	23.32	16.05	5.66	5.18	114,732.25 (112,977.37 to 116,487.12)	1,915.56 (1,875.65 to 1,955.47)	98,324.48 (96,009.08 to 99,432.24)	1,551.83 (1,523.78 to 1,582.43)
2018	7,053	89.55	29.36	21.20	23.73	16.12	5.63	5.08	116,894.01 (115,208.91 to 118,579.10)	2,157.60 (2,065.57 to 2,249.63)	100,793.86 (98,645.16 to 101,811.98)	1,630.57 (1,608.85 to 1,654.05)
2019	7,265	89.37	29.10	20.83	23.65	15.84	5.45	4.99	116,187.58 (114,586.56 to 117,788.60)	2,049.53 (1,995.52 to 2,103.54)	100,000.00 (99,716.43 to 100,939.69)	1,646.15 (1,627.36 to 1,673.70)

a All dialysis facilities in the US. The existence of a facility*year is jointly defined by the CMS facility annual survey, patient treatment history records, and Medicare certification and termination dates. Physician owners include both individual owners and physicians that associate with an institutional owner. Physicians associated with institutional owners that are linked to more than 50 physicians were not considered owners due to their small ownership stake. Annual compensation is Winsorized at 1- ($11,356) and 99- ($397,723) percentiles in the pooled sample from years 2005 – 2019. All dollar amounts are inflation-adjusted to 2019 values.

© 2025 Xia X et al. *JAMA Health Forum*.

eTable 4: DaVita Physician-owned Facility Validation[a]

Year	2005	2006	2007	2008	2009	2010	2011	2012	2013	2014	2015	2016	2017	2018	2019
This Paper	70	89	113	149	176	208	273	350	404	483	521	577	653	695	707
This Paper (Individual NPIs Only)	54	69	94	115	139	166	212	279	325	389	416	470	536	566	582
Official DaVita Count	-	-	-	259	287	292	326	369	403	485	485	533	587	671	

a Our baseline analysis identifies physician owners using both individual owners and institutional owners from ESRD PECOS (see eMethod A.2).

© 2025 Xia X et al. *JAMA Health Forum*.

eTable 5: Distribution of Chains Owned/Directed Among Owners/Medical Directors, 2019[a]

	**Direct at least 1 DaVita Facility**	**Direct at least 1 Fresenius Facility**	**Direct at least 1 Other Chain Facility**	**Direct at least 1 Independent Facility**	**Own at least 1 DaVita facility**	**Own at least 1 Fresenius facility**	**Own at least 1 Other Chain facility**	**Own at least 1 independent facility**
Mean DaVita Facilities	1.39 (1.37 to 1.42)	0.06 (0.05 to 0.07)	0.09 (0.06 to 0.11)	0.17 (0.13 to 0.21)	2.80 (2.65 to 2.94)	0.93 (0.74 to 1.12)	1.09 (0.87 to 1.31)	0.60 (0.48 to 0.73)
Mean Fresenius Facilities	0.07 (0.05 to 0.08)	1.39 (1.36 to 1.42)	0.09 (0.05 to 0.13)	0.09 (0.06 to 0.12)	0.47 (0.36 to 0.57)	4.19 (3.79 to 4.60)	0.49 (0.33 to 0.65)	1.01 (0.79 to 1.24)
Mean Other Chain Facilities	0.03 (0.02 to 0.03)	0.02 (0.01 to 0.03)	1.31 (1.27 to 1.36)	0.03 (0.01 to 0.05)	0.24 (0.20 to 0.27)	0.17 (0.13 to 0.20)	2.00 (1.86 to 2.14)	0.30 (0.19 to 0.42)
Mean Independent Facilities	0.05 (0.03 to 0.06)	0.03 (0.02 to 0.04)	0.03 (0.01 to 0.04)	1.20 (1.15 to 1.24)	0.12 (0.10 to 0.14)	0.23 (0.18 to 0.28)	0.18 (0.14 to 0.22)	1.62 (1.53 to 1.70)

a Physicians associated with companies that are linked to more than 50 physicians were not considered owners due to their small ownership stake. Each observation is a physician-month. 95% confidence intervals are in parentheses.

© 2025 Xia X et al. *JAMA Health Forum*.

eTable 6: Freestanding Dialysis Facility Medical Director Compensation Summary Statistics by Chain, 2005 and 2019[a]

	**Mean, $**	**95% CI of Mean, $**	**Median, $**	**95% CI of Median, $**	**Std Dev, $**	**No.**
2005						
All Facilities	120,829.83	117,684.98 to 123,974.68	94,251.43	91,628.09 to 97,962.54	91,569.88	3,257
DaVita	111,043.72	106,755.77 to 115,331.67	91,967.14	87,317.67 to 97,060.75	75,753.66	1,199
Fresenius	161,921.58	155,285.63 to 168,557.52	134,674.16	125,713.34 to 143,515.45	112,086.14	1,096
Other Chains	90,033.89	85,473.19 to 94,594.58	75,643.31	70,885.20 to 79,435.29	61,695.41	703
Independent	75,835.76	68,880.46 to 82,791.06	61,551.42	54,054.75 to 68,725.00	57,109.64	259
2019						
All Facilities	116,187.58	114,586.56 to 117,788.60	100,000.00	99,716.43 to 100,939.69	66,951.69	6,718
DaVita	108,099.28	106,454.47 to 109,744.10	100,000.00	99,758.32 to 102,326.27	44,832.01	2,854
Fresenius	135,582.56	132,339.06 to 138,826.06	110,392.00	107,149.13 to 113,784.98	85,348.98	2,660
Other Chains	98,670.94	95,384.26 to 101,957.63	90,000.00	85,000.00 to 95,000.00	49,032.55	855
Independent	77,419.84	71,857.46 to 82,982.23	67,650.00	61,709.50 to 75,000.00	53,017.30	349

a Medical director compensation for freestanding dialysis centers from Healthcare Cost Report Information System (HCRIS) Dataset. Not all freestanding facilities reported their medical director compensation (see Figure 2). Annual compensation is Winsorized at 1- ($11,356) and 99- ($397,723) percentiles in the pooled sample from years 2005 – 2019. All dollar amounts are inflation-adjusted to 2019 values.

© 2025 Xia X et al. *JAMA Health Forum*.

eTable 7: Freestanding Dialysis Facility Medical Director Compensation *per Patient* Summary Statistics by Chain, 2005 and 2019[a]

	Mean, $	95% CI of Mean, $	Median, $	95% CI of Mean, $	Std Dev, $	No.
2005						
All Facilities	1,797.08	1,721.39 to 1,872.77	1,540.06	1,507.94 to 1,567.01	2,160.53	3,130
DaVita	1,663.75	1,474.85 to 1,852.66	1,428.79	1,382.73 to 1,461.56	3,252.75	1,139
Fresenius	2,346.12	2,278.23 to 2,414.02	2,138.67	2,064.43 to 2,220.43	1,127.81	1,060
Other Chains	1,403.86	1,349.24 to 1,458.47	1,288.21	1,229.56 to 1,337.98	732.51	691
Independent	1,137.08	996.05 to 1,278.10	904.79	815.21 to 993.01	1,114.69	240
2019						
All Facilities	2,049.53	1,995.52 to 2,103.54	1,646.15	1,627.36 to 1,673.70	2,218.80	6,483
DaVita	2,185.13	2,078.13 to 2,292.13	1,633.47	1,599.15 to 1,671.92	2,867.94	2,760
Fresenius	2,054.98	2,003.58 to 2,106.38	1,791.47	1,760.61 to 1,830.70	1,332.03	2,580
Other Chains	1,757.68	1,638.53 to 1,876.84	1,470.74	1,429.37 to 1,527.86	1,755.68	834
Independent	1,580.51	1,304.67 to 1,856.35	1,001.72	885.36 to 1,189.66	2,473.90	309

a Medical director compensation for freestanding dialysis centers from Healthcare Cost Report Information System (HCRIS) Dataset. Not all freestanding facilities reported their medical director compensation (see Figure 2). Annual compensation is Winsorized at 1- ($11,356) and 99- ($397,723) percentiles in the pooled sample from years 2005 – 2019. All dollar amounts are inflation-adjusted to 2019 values.

© 2025 Xia X et al. *JAMA Health Forum*.

eTable 8: Effects on Medicare Price for Hemodialysis across Markets, OLS regressions

Indicators of HSA Characteristics (1 if true)	**Hemodialysis Price ($)** Coefficient (95% CI)	**Hemodialysis Price ($)** Coefficient (95% CI)	**Hemodialysis Price ($)** Coefficient (95% CI)
In HSA with No Top-5 Chain	9.30 (3.55 to 15.05)	6.60 (0.31 to 12.88)	6.92 (0.66 to 13.17)
In HSA with 1 Top-5 Chain (Normalized Baseline)	0 (NA)	0 (NA)	0 (NA)
In HSA with 2 Top-5 Chains	0.34 (-2.56 to 3.24)	1.13 (-1.88 to 4.14)	0.67 (-2.40 to 3.74)
In HSA with 3 Top-5 Chains	-7.54 (-12.48 to -2.60)	-6.62 (-11.74 to -1.50)	-7.40 (-12.73 to -2.07)
In HSA with 4 Top-5 Chains	-8.65 (-15.33 to -1.98)	-7.79 (-14.46 to -1.12)	-8.69 (-15.54 to -1.85)
In HSA with Independent Facility		3.77 (0.57 to 6.96)	3.80 (0.61 to 6.98)
In HSA with Joint Ventures			2.36 (-0.08 to 4.81)
Constant	211.67 (209.02 to 214.33)	209.60 (206.23 to 212.97)	208.59 (205.29 to 211.88)
Observations	28,478,017	28,478,017	28,478,017
Month Fixed Effects	YES	YES	YES
Number of Facilities Fixed Effects	YES	YES	YES

a Monthly Medicare price from USRDS Medicare claims, from Jan 2012 to Dec 2020. Each observation is a patient-month. Top-5 chains are DaVita, Fresenius, United States Renal Care, American Renal Associates, and Dialysis Clinic, Inc.

© 2025 Xia X et al. *JAMA Health Forum*.

eTable 9: Effects on Standardized 1-Year Mortality Rate (SMR), OLS regressions[a]

Indicators of HSA Characteristics (1 if true)	**SMR** Coefficient (95% CI)	**SMR** Coefficient (95% CI)	**SMR** Coefficient (95% CI)
In HSA with No Top-5 Chain	0.00<br>(-2.39 to 2.40)	-1.05<br>(-3.68 to 1.57)	-1.05<br>(-3.67 to 1.56)
In HSA with 1 Top-5 Chain (Normalized Baseline)	0<br>(NA)	0<br>(NA)	0<br>(NA)
In HSA with 2 Top-5 Chains	1.02<br>(-0.78 to 2.81)	1.41<br>(-0.44 to 3.26)	1.57<br>(-0.31 to 3.44)
In HSA with 3 Top-5 Chains	1.80<br>(-0.91 to 4.51)	2.28<br>(-0.51 to 5.08)	2.69<br>(-0.20 to 5.57)
In HSA with 4 Top-5 Chains	3.34<br>(-1.35 to 8.04)	3.93<br>(-0.86 to 8.71)	4.50<br>(-0.31 to 9.31)
In HSA with Independent Facility		1.48<br>(-.06 to 3.02)	1.38<br>(-0.16 to 2.91)
In HSA with Joint Ventures			-1.43<br>(-2.87 to 0.01)
Constant	106.84<br>(106.01 to 107.66)	106.51<br>(105.62 to 107.41)	106.85<br>(105.90 to 107.79)
Observations	30541	30541	30541
Month Fixed Effects	YES	YES	YES
Number of Facilities Fixed Effects	YES	YES	YES

a Data from USRDS, 2012 to 2020. Each observation is a facility-year. Top-5 chains are DaVita, Fresenius, United States Renal Care, American Renal Associates, and Dialysis Clinic, Inc. Standard errors in parentheses. The standardized 1-year mortality rate (SMR) is calculated based on the one-year mortality rate of patients under the care of the facility in each year, weighted by patient-days spent in that facility during that year. The normalization is conducted by sex (male, female)-age (<18, 18-44, 45-64, 65-84, >84) bins. The SMR shown is the facility-year's actual mortality rate divided by the expected mortality rate normalized on the national sample.

© 2025 Xia X et al. *JAMA Health Forum*.

eTable 10: Facility Count Shares by Chains from 2005 to 2019 (%)[a]

Year	DaVita	Fresenius	USRC	DCI	ARA	Other Chains	Independent
2005	30.90	28.19	0.00	4.85	0.99	14.71	20.35
2006	30.90	36.32	0.00	4.67	1.20	7.29	19.62
2007	31.13	35.74	0.00	4.57	1.40	7.54	19.63
2008	32.05	35.62	0.06	4.47	1.56	8.08	18.16
2009	32.37	35.37	0.78	4.30	1.59	9.75	15.83
2010	33.28	35.21	1.66	4.20	1.66	8.97	15.02
2011	35.29	35.18	1.74	4.07	1.97	7.82	13.93
2012	36.77	38.21	1.49	3.97	2.25	3.80	13.51
2013	37.32	37.76	2.30	3.81	2.43	3.24	13.13
2014	37.87	37.05	2.36	3.85	2.72	3.38	12.78
2015	38.05	37.06	2.57	3.74	3.04	3.30	12.23
2016	39.05	37.66	4.05	3.66	3.15	1.76	10.67
2017	39.84	37.38	4.46	3.54	3.29	1.17	10.31
2018	39.77	37.45	4.32	3.59	3.32	1.11	10.45
2019	39.81	37.27	4.32	3.52	3.34	1.10	10.63

a Market share defined by facility counts.

© 2025 Xia X et al. *JAMA Health Forum*.

eTable 11: National Population Shares by HSA Types from 2005 to 2019 (%)[a]

Year	With only DaVita	With only Fresenius	With only DaVita or Fresenius	With Facilities Other than DaVita or Fresenius	With Any Facility	With No Facility
2005	8.68	8.62	21.56	49.11	70.68	29.32
2006	8.46	11.26	26.78	46.25	73.03	26.97
2007	8.49	11.17	26.92	48.64	75.55	24.45
2008	8.25	10.66	26.28	50.65	76.93	23.07
2009	8.07	10.89	26.44	52.19	78.64	21.36
2010	8.28	10.13	26.86	52.84	79.70	20.30
2011	8.63	10.06	28.34	52.68	81.03	18.97
2012	9.09	10.67	32.01	50.39	82.39	17.61
2013	9.26	10.25	31.85	51.95	83.79	16.21
2014	9.17	9.80	32.32	52.68	85.00	15.00
2015	9.03	9.77	32.44	53.51	85.95	14.05
2016	9.33	9.47	33.48	53.21	86.69	13.31
2017	8.95	8.94	33.49	53.81	87.30	12.70
2018	8.55	8.49	32.63	55.24	87.87	12.13
2019	8.27	8.24	32.54	55.76	88.31	11.69

a National population shares by HSA facility accessibility. ZIP-level population data are from 2013 5-year ACS for years 2003-2013, and from 2021 5-year ACS for years 2014-2019. ZIP-HSA crosswalks are from Dartmouth Atlas Data.

© 2025 Xia X et al. *JAMA Health Forum.*

eTable 12: Comparison of Facility Counts across Market Types

	Any Facilities (N)			Independent Facilities (N)			Any Chain Facilities (N)		
	Mean	Median	SD	Mean	Median	SD	Mean	Median	SD
2005									
HSA with No Top-5 Chain	1.50	1.00	1.07	0.90	1.00	1.10	0.61	0.00	0.85
HSA with 1 Top-5 Chain	2.79	2.00	3.13	0.58	0.00	1.37	2.21	1.00	2.32
HSA with 2 Top-5 Chain	13.84	10.00	13.73	2.04	1.00	3.01	11.80	8.00	11.21
HSA with 3 Top-5 Chain	21.53	22.00	9.99	3.30	1.00	3.73	18.23	17.00	9.04
2019									
HSA with No Top-5 Chain	1.60	1.00	1.26	1.53	1.00	1.31	0.08	0.00	0.32
HSA with 1 Top-5 Chain	2.02	1.00	1.79	0.29	0.00	0.94	1.74	1.00	1.32
HSA with 2 Top-5 Chain	7.73	5.00	7.09	0.86	0.00	2.12	6.87	5.00	6.21
HSA with 3 Top-5 Chain	23.17	16.00	18.44	2.94	1.00	4.37	20.23	15.00	15.42
HSA with 4 Top-5 Chain	59.88	37.00	47.77	8.38	1.00	13.39	51.50	36.00	34.89

© 2025 Xia X et al. *JAMA Health Forum*.

eFigure 1: Study Flowchart

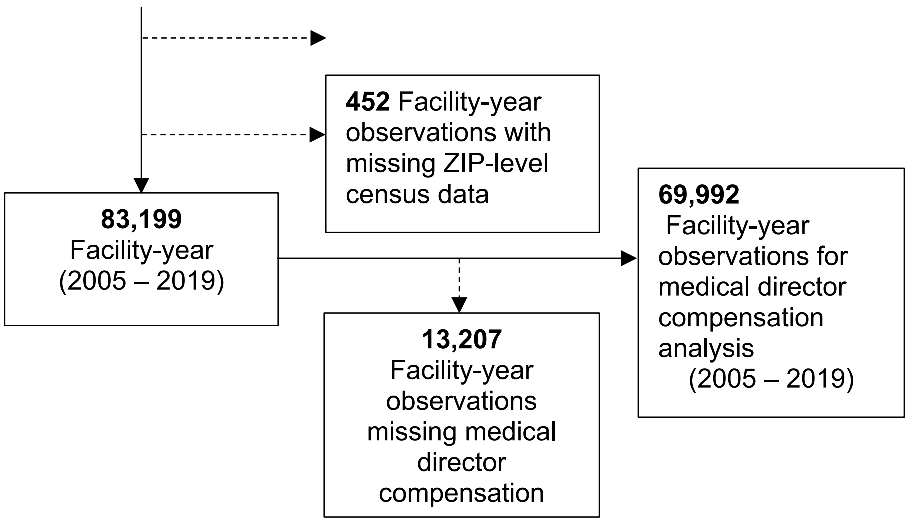

© 2025 Xia X et al. *JAMA Health Forum*.

eFigure 2: Share of National Population by HSA Types among Existing Markets in 2025, 2005 to 2019 (%)[a]

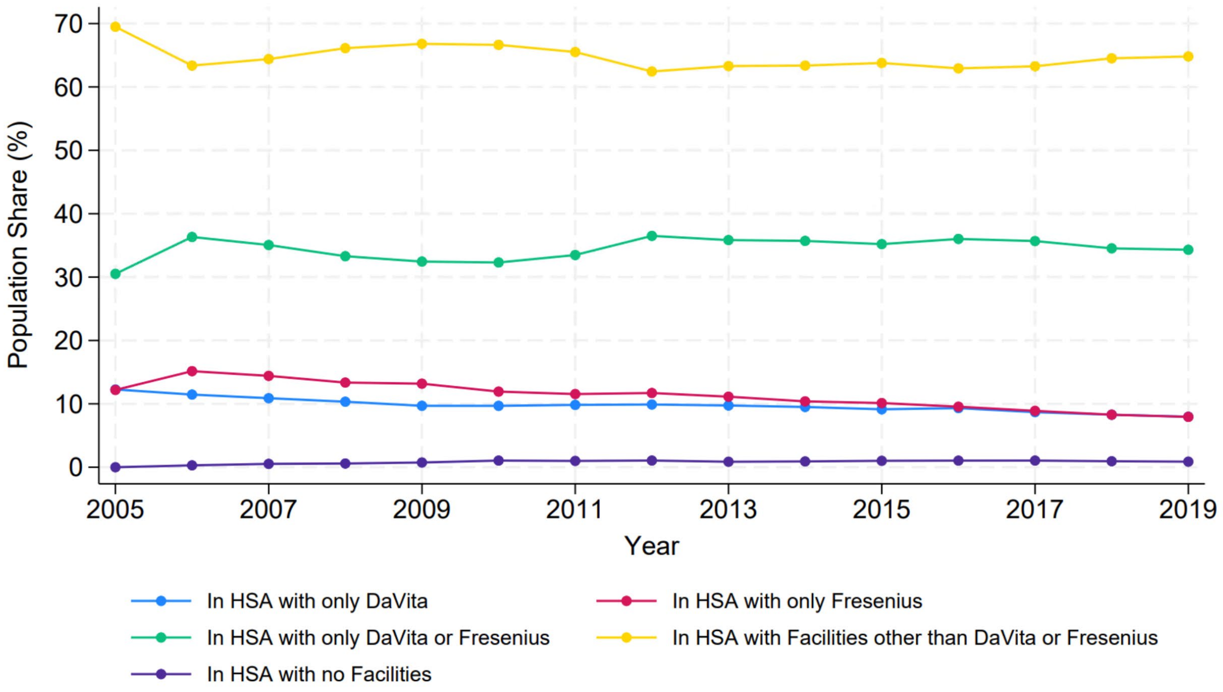

a Share of the national population living in HSAs with different types of facility chain configuration and with existing facilities in 2005.

© 2025 Xia X et al. *JAMA Health Forum*.

eFigure 3: Share of Entries into Markets with No Existing Facilities[a]

(a) Share of New Entry HSAs without Existing Facility

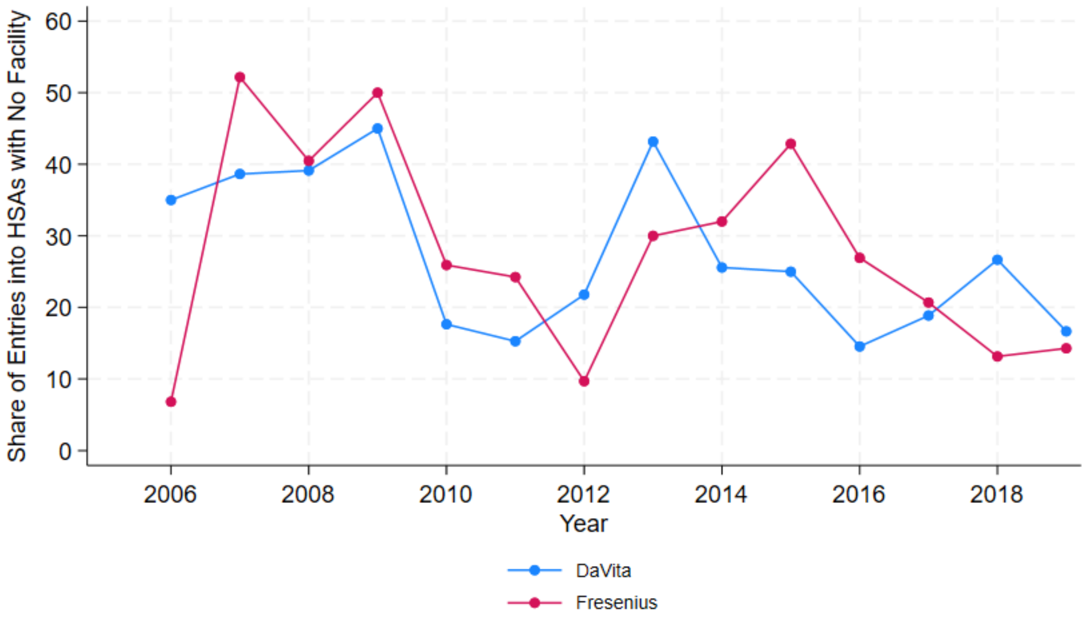

(b) Share of New Entry HSA Population without Existing Facility

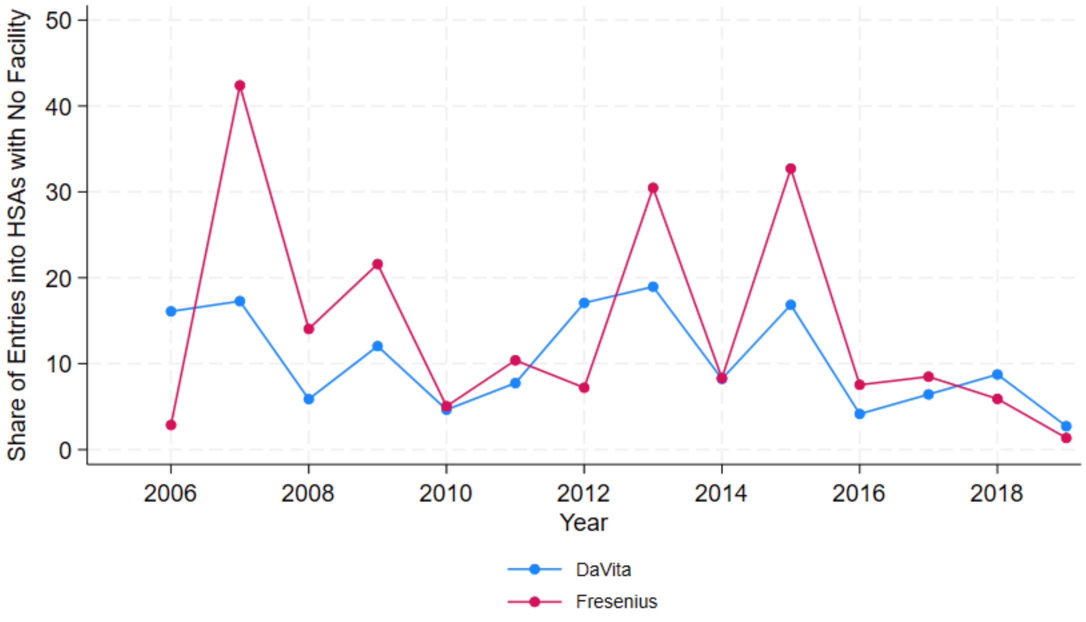

a Panel (a) shows the number of HSAs where the chain had new facilities (via either De Nevo entry or acquisition) and the HSA had no existing facility, divided by the number of HSAs the number of HSAs where the chain had new facilities (via either De Nevo entry or acquisition). Panel (b) wights HSAs in (a) by population.

© 2025 Xia X et al. *JAMA Health Forum*.

We can see that, among all the HSAs the chain had new facilities in, around 30% of the markets had no existing facility, accounting for 10 – 20% of the population they entered. These entries do help prove patients with dialysis facilities in their own market, but their welfare effect on patients has its nuances. This is not necessarily beneficial to patients if it crowds out the existing provision of home hemodialysis or peritoneal dialysis, in addition to pre-empting potential, maybe cheaper, competitors. We leave it for future work to better address the general dynamic effects of this type of market expansion.

© 2025 Xia X et al. *JAMA Health Forum*.

eFigure 4: State-level Population Shares in HSAs with only DaVita or Fresenius (%)

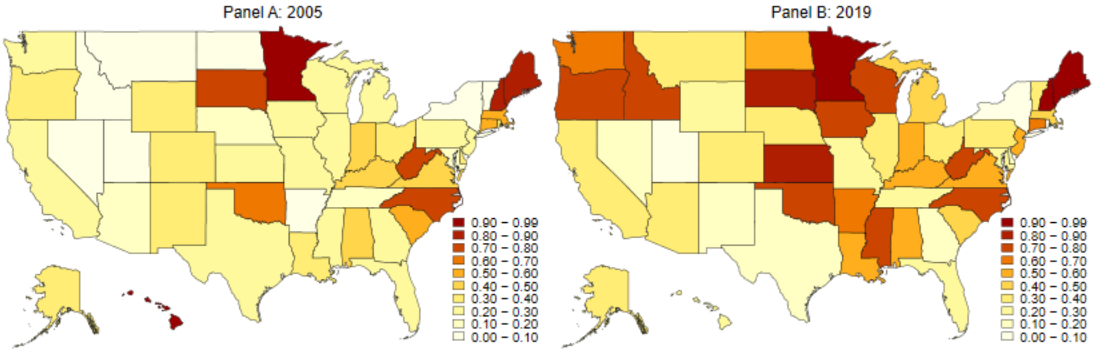

© 2025 Xia X et al. *JAMA Health Forum*.

eFigure 5: Trends of Market Concentration[a]

(a) Share of Dialysis Facilities Count by Chain Ownership, 2000 to 2019 (%)

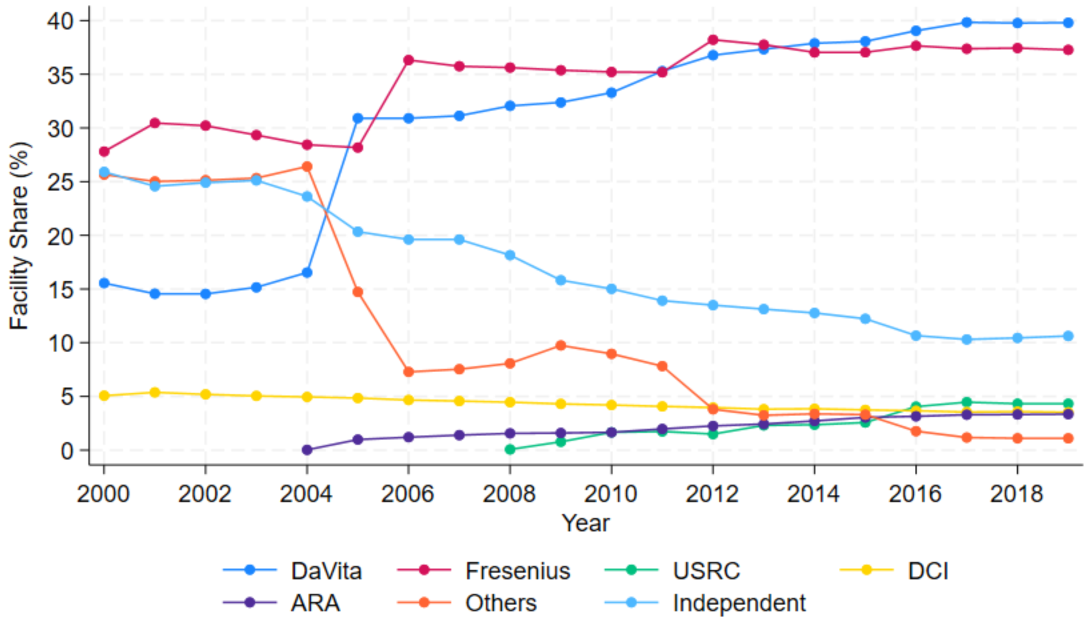

(b) Share of National Population by Residing HSA's Chain Configuration, 2000 to 2019 (%)

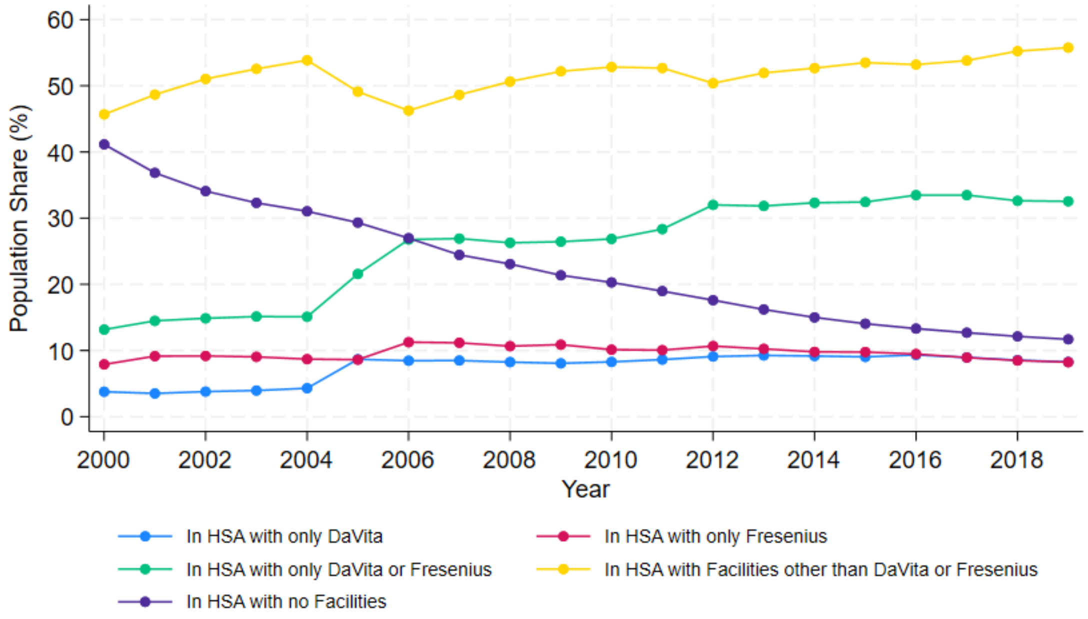

© 2025 Xia X et al. *JAMA Health Forum*.

eReferences

1. Noridian Medicare. HPSA physician specialties. Accessed April 11, 2024. https://med.noridianmedicare.com/web/jeb/topics/incentive-programs/hpsa-hsip/hpsa-physician-specialties
2. DaVita Kidney Care. Joint ventures and acquisitions. Accessed April 8, 2024. https://www.davita.com/physicians/partnerships/joint-ventures-acquisitions
3. Fowler A, Grabowski D, Gambrel R, Huskamp H, Stevenson D. Corporate investors increased common ownership in hospitals and the postacute care and hospice sectors. Health Aff (Millwood). 2017;36(9):1547-1555. doi:10.1377/hlthaff.2017.0207
4. League RJ, Eliason P, McDevitt RC, Roberts JW, Wong H. Variability in prices paid for hemodialysis by employer-sponsored insurance in the US from 2012 to 2019. JAMA network open. 2022 Feb 1;5(2):e220562-.

© 2025 Xia X et al. *JAMA Health Forum*.
