# Supplementary material for: Financial Ties, Market Structure, Commercial Prices, and Medical Director Compensation in Dialysis
Source: JAMA Health Forum. 2025 Jun 18;6(6):e252659. doi: 10.1001/jamahealthforum.2025.2659 (PMC12177639; doi:10.1001/jamahealthforum.2025.2659)
Supplement: Supplement 2. — Data Sharing Statement [file jamahealthforum-e252659-s002.pdf]

Data Sharing Statement

Xia. Financial Ties, Market Structure, Commercial Prices, and Medical Director Compensation in Dialysis. *JAMA Health Forum*. Published June 18, 2025.
doi:10.1001/jamahealthforum.2025.2659

Data
Data available: No

Additional Information
Explanation for why data not available: Authors are unable to share the data due to restrictions in the data user agreements (DUAs). Medicare dialysis claim data can be applied via United States Renal Data System (USRDS); PECOS data can be applied via Freedom of Information Act (FOIA) requests; OpenCorporates data can be applied on their website.
